# Protective effect of dexamethasone on 5-FU-induced oral mucositis in hamsters

**DOI:** 10.1371/journal.pone.0186511

**Published:** 2017-10-23

**Authors:** Susana Barbosa Ribeiro, Aurigena Antunes de Araújo, Raimundo Fernandes de Araújo Júnior, Gerly Anne de Castro Brito, Renata Carvalho Leitão, Maisie Mitchele Barbosa, Vinicius Barreto Garcia, Aldo Cunha Medeiros, Caroline Addison Carvalho Xavier de Medeiros

**Affiliations:** 1 Post Graduation Program in Biotechnology RENORBIO, UFRN, Natal, RN, Brazil; 2 Post Graduation Program Public Health/Post Graduation Program in Pharmaceutical Science/Department of Biophysics and Pharmacology, UFRN, Natal, RN, Brazil; 3 Post Graduation Program in Functional and Structural Biology/Post Graduation Program Health Science/Department of Morphology, UFRN, Natal, RN, Brazil; 4 Post Graduation Program of Morphological Science/Department of Morphology, UFC, Fortaleza, CE, Brazil; 5 Post Graduation Program of Morphological Science/Department of Morphology, UFC, Fortaleza, CE, Brazil; 6 Post Graduation Program in Biological Sciences, UFRN, Natal, RN, Brazil; 7 Post Graduation Program Health Science, UFRN, Natal, RN, Brazil; 8 Post Graduation Program Health Science, Department of Surgery, UFRN, Natal, RN, Brazil; 9 Post Graduation Program in Biotechnology RENORBIO/ Post Graduation Program in Biological Sciences/ Department of Biophysics and Pharmacology, UFRN, Natal, RN, Brazil; Augusta University, UNITED STATES

## Abstract

Oral mucositis (OM) is an important side effect of cancer treatment, characterized by ulcerative lesions in the mucosa of patients undergoing radiotherapy or chemotherapy, which has marked effects on patient quality of life and cancer therapy continuity. Considering that few protocols have demonstrated efficacy in preventing this side effect, the aim of this study was to examine the effect of dexamethasone (DEX) on OM induced by 5-fluorouracil (5-FU) in hamsters by studying signaling pathways. OM was induced in hamsters by 5-FU followed by mechanical trauma (MT) on day 4. On day 10, the animals were euthanized. The experimental groups included saline, MT, 5-FU, and DEX (0.25, 0.5, or 1 mg/kg). Macroscopic, histopathological, and immunohistochemical analyses as well as immunofluorescence experiments were performed on the oral mucosa of the animals. The oral mucosal samples were analyzed by enzyme-linked immunosorbent assays, and quantitative real-time polymerase chain reaction (qPCR). DEX (0.5 or 1 mg/kg) reduced inflammation and ulceration of the oral mucosa of hamsters. In addition, DEX (1 mg/kg) reduced the cytokine levels of tumor necrosis factor (TNF)-α, interleukin (IL)-1β, and macrophage migration inhibitory factor (MIF). DEX (1 mg/kg) also reduced the immunoexpression of cyclooxygenase (COX)-2, matrix metalloproteinase (MMP)-2, transforming growth factor (TGF)-β, MIF, Smad 2/3, Smad 2/3 phosphorylated and NFκB p65 in the jugal mucosa. Finally, DEX (1 mg/kg) increased interleukin-1 receptor-associated kinase 3 (IRAK-M), glucocorticoid-induced leucine zipper (GILZ), and mitogen-activated protein kinase (MKP1) gene expression and reduced NFκB p65 and serine threonine kinase (AKt) gene expression, relative to the 5-FU group. Thus, DEX improved OM induced by 5-FU in hamsters.

## Introduction

Oral mucositis (OM) is a severe adverse effect that occurs during cancer treatment. It is characterized by painful ulcers in the oral mucosa, negatively impacting the quality of life of patients and impairing anticancer therapy [1]. The prevalence of OM is higher for patients undergoing radiotherapy, for those receiving treatment for head and neck cancers (29–66%), and for those receiving high doses of chemotherapy before transplantation of hematopoietic stem cells (75–85%) [2]. The damage caused by chemotherapy or radiotherapy begins in the endothelial tissue of the submucosa, leading to apoptosis of the submucosal cells. Therefore, macrophages and transcription factors are activated [3] [4]. The nuclear transcription factor kappa B (NFκB) is the most studied transcription factor in OM because it is an important regulator of proinflammatory cytokine expression (e.g., tumor necrosis factor (TNF)-α and interleukin (IL)-1β, cyclooxygenase-2 (COX-2), as well as matrix metalloproteinases (MMPs). TNF-α stimulates the production of NFκB, resulting in tissue injury and cell death [5]. NFκB is found in the cytoplasm bound to inhibitory κB proteins (IκBs) and is activated after degradation of IκB, resulting in translocation of the predominant dimer of the canonical pathway p50/p65 to the cell nucleus. In the nucleus, the dimer controls the transcription of various inflammatory genes, survival, and cell division [6].

Therapeutic interventions that reduce the expression of proinflammatory cytokines and NFκB have been shown to be effective for OM [7]. Management of oral mucositis has been largely palliative to date. Few protocols have demonstrated efficacy in the prevention of this inflammatory condition that impairs the patient’s quality of life and may drastically affect cancer treatment [8]. Dexamethasone (DEX) is an anti-inflammatory agent that is widely used in clinical practice [9]. The protective effect of DEX on OM induced by chemotherapy has been described in the literature [10]. However, the molecular mechanism involved in its protective effect on OM has not been elucidated. The effects of DEX are mediated by the glucocorticoid receptor (GR), which regulates gene expression either positively or negatively. GR can regulate gene expression by binding to the glucocorticoid response elements (GREs) in the target gene promoter region or by interacting with transcription factors such as NFκB, thus interfering with gene expression [11]. Glucocorticoids modulate various signaling pathways, such as glucocorticoid-induced leucine zipper (GILZ) [12], interleukin-1 receptor-associated kinase 3 (IRAK-M) [13], MAPK phosphatase 1 (MKP1) [14], and macrophage migration inhibitory factor (MIF) [15]. The aim of this study was investigate the molecular mechanisms involved in the anti-inflammatory effects of DEX on OM induced by 5-fluorouracil (5-FU) in hamsters.

## Materials and methods

### OM model and experimental groups

*Golden sirian (Mesocricetus auratus*) male hamsters, weighing between 130 and 200 g, were used in the present study. This project was approved by the Animal Experiment Ethics Committee of Federal University of Rio Grande do Norte, protocol number (071/2014). 5-FU was administered to induce OM on days 1 and 2 of the experiment (60 and 40 mg/kg, i.p., respectively). On day 4, after anesthesia with ketamine (70 mg/kg, i.p.) and xylazine (10 mg/kg, i.p.), the animals were subjected to mechanical trauma (MT), following an established protocol adapted from Sonis et al (1990) [16]. MT consists of excoriations in the oral mucosa of the animal, with a 25x7 mm gauge needle, to mimic the clinical effect of oral trauma and irritation such as ill-fitting dentures, orthodontic bands, and others appliances. OM is induced with 5-FU followed by MT [17,18]. Finally, on day 10, the animals were euthanized with thiopental (80 mg/kg, i.p.) [17]. The treated groups were divided into 3 subgroups that differed only in the concentration of DEX (Aché Pharmaceutical, Brazil 0.25, 0.5, or 1 mg/kg, i.p.), dissolved in saline solution [19] and administered 1 hour before the first 5-FU injection and, daily, once a day, until the euthanasia, on day 10. All treated animals received 5-FU and mechanical trauma in the right cheek pouch mucosa. The control groups were divided into 3 subgroups: a group of animals not subjected to OM, which received intraperitoneal injections of saline, once a day, for 10 days (Saline); hamsters that received only mechanical trauma and daily injections of saline (i.p.) for 10 days (MT) and hamsters subjected to oral mucositis by 5-FU and MT, as described above, and received, daily, until the euthanasia, intraperitoneal injections of saline, starting 1 hour before the first 5-FU (5-FU). There were at least 6 animals in each experimental group. The experiments were pooled.

Macroscopic and histopathological analysis of the oral mucosa were performed on the jugal mucosa. In addition, cytokine quantification of TNF-α and IL-1β; immunohistochemistry for COX-2, TGF-β, and MMP-2; immunofluorescence for MIF, Smad 2/3, Smad 2/3 phosphorylated protein and NFκB p65; and quantitative real-time polymerase chain reaction (qPCR) for IRAK-M, GILZ, MKP1, NFκB p65, and serine threonine kinase (AKt) were carried out.

### Macroscopic and histopathological analysis

The macroscopic and histopathological analysis of the hamster jugal mucosa were performed using the methodology previously described [17]. The jugal mucosa of the hamsters was exposed, photographed, and scored as follows: 0, healthy mucosa and no evidence of erosion or vasodilation; 1, presence of erythema and no evidence of mucosal erosion; 2, severe erythema, vasodilatation, and superficial erosion; 3, presence of ulcers in one or more faces of the mucosa, affecting no more than 25% of the area, severe erythema, and vasodilatation; 4, ulcers in about 50% of the area of the jugal mucosa; 5, completely ulcerated jugal mucosa that made it impossible to expose the tissue.

For histopathology, the oral mucosa was fixed in 10% buffered formaldehyde solution for 24 h, embedded in paraffin, and stained with hematoxylin and eosin. The samples were analyzed microscopically and scored as follows: 1, epithelium and connective tissue without vasodilation, absent or discrete cellular infiltration, and absence of bleeding, edema, ulcers, and abscesses; 2, discrete vasodilatation, areas of re-epithelialization, discrete cell infiltration with a high number of mononuclear leukocytes, and absence of hemorrhage, edema, ulcers, and abscesses; 3, moderate vasodilatation, epithelial hydropic degeneration (vacuolization), moderate cellular infiltration with a predominance of polymorphonuclear leukocytes, presence of hemorrhagic areas, edema, and occasional small ulcers, and absence of abscesses; 4, marked vasodilation, marked cell infiltration with a high number of polymorphonuclear leukocytes, presence of hemorrhagic areas, edema, and eventual ulceration, and absence of abscesses; 5, severe vasodilatation and inflammatory infiltration with neutrophils, abscesses, and extensive ulcers.

### Cytokine quantification

Cytokine quantification was performed using an enzyme-linked immunosorbent assay kit (R&D Systems, Minneapolis, Minnesota, USA) by reading the absorbance at 450 nm [20]. A total of 100 mg of the jugal mucosa were macerated in 600 μL of phosphate-buffered saline (PBS), yielding a homogenate that was incubated for 2 h with TNF-α or IL-1β-capture antibodies. The plates were washed three times with washing buffer and then incubated with biotinylated secondary antibody. The plates were washed with washing buffer before the addition of diluted streptavidin solution and developing solution (H_2_O_2_ and tetramethylbenzidine).

### Immunohistochemistry

Immunohistochemistry for MMP-2, COX-2, and TGF-β was performed according to previously described methodology [21]. The avidin–biotin–peroxidase method was used. The jugal mucosa tissue samples were deparaffinized using different xylol concentrations. The samples were incubated with primary antibody (1:400) for 12 h at 4°C and then with biotinylated secondary antibody. The slides were stained with 3,3ʹ-diaminobenzidine tetrahydrochloride and with Harris hematoxylin. The Santa Cruz Biotechnology primary antibodies used were: mouse polyclonal anti-MMP2 (1:400; sc-13595); goat polyclonal anti-COX2 (1:400; sc-1746) and rabbit polyclonal anti-TGF-β (1:400; sc-146). Before starting the method, tests of dilution with white control were performed to choose the best dilution and pattern the label to the samples of hamster. The scores were performed using the semi-quantitative method where five fragments (05 images) of tissue of five animals per group were evaluated. The intensity and the quantity of labeled cells were graduated per quadrants (100uM) whose the scores one (1) were to absence of intensity and quantity of the labeling, (2) two to weak intensity and quantity, (3) three to moderate intensity and quantity and (4) four to intense intensity and quantity of the labeling.

### Immunofluorescence

Immunofluorescence was performed according to previously described methodology [22]. The hamster jugal mucosa samples (n = 5, per group) were deparafinized and washed with various concentrations of ethanol and PBS. Samples were incubated at 4°C overnight with rabbit polyclonal anti-MIF primary antibody (1:400; sc 20121, Santa Cruz Biotechnology), mouse monoclonal anti-NFkB primary antibody (1:200; sc-8008, Santa Cruz Biotechnology), rabbit polyclonal anti-SMAD primary antibody (1:400; sc-8332, Santa Cruz Biotechnology) and rabbit polyclonal anti-pSMAD primary antibody (1:400; sc-11769-R, Santa Cruz Biotechnology). Then, the slides were washed in PBS/0.2% triton X-100 for 5 min, and incubated with Alexa Fluor 488 (1:500 in 1% BSA) and and were mounted with Fluoroshield Mounting Medium with DAPI (abcam®, UK). The quantitative analysis of the fluorescence intensity was determined from digital images of at least 3 different areas of each section (five animals per group), on 100x magnification, using the Zeiss ZEN lite blue edition software.

### qPCR

The mRNA expression for IRAK-M, GILZ, MKP1, NFκB p65, and AKt in the hamster jugal mucosa was determined. Trizol (Life Technologies, Carlsbad, CA, USA) and SYBR Green Master Mix were used according to previously described methodology [23]. Isolation of the total RNA was performed with the total SV Total RNA isolation system (Promega Corporation, USA), and the total RNA concentration was determined by the optical density at 260 nm. For cDNA synthesis, 10 μL (5 μg) of total RNA, 2 μL of 10× RT buffer, 0.8 μL of 25× dNTP Mix (100 mM), 2.0 μL of 10× RT Random primer, 1 μL of MultiScribe Reverse Transcriptase, 4.2 μL of Nuclease-free H_2_O (High-Capacity cDNA Reverse Transcription Kit, Foster City, CA, USA) in a total volume of 20 μL were used. The plates were placed in the Step One Plus thermal cycler (Applied Biosystems, USA), according to the manufacturer’s instructions. For each 1× 5 mL Power up Syber Green Master Mix, 2.0 μL of cDNA was added to a final volume of 10 μL. The PCR conditions were as follows: 95°C for 5 min and 40 cycles of 30 s at 95°C, 30 s at 52–60°C (target-based), and 60 s at 72 ºC. The specificity of the resulting PCR products was confirmed by means of melting curves. The relative quantitative fold change compared with the control (saline group) was calculated using the comparative Ct method, where Ct is the cycle number at which fluorescence first exceeds the threshold. The Ct values for each sample were obtained by subtracting the value for the GADPH Ct from the target gene Ct value. The specificity of the resulting PCR products was confirmed by melting curves.

Sequence-specific oligonucleotide primers were designed using Primer Express software version 3.0.1 (Applied Biosystems). The initiator sequences encode GILZ (also known as TSC22 domain family member 3, Tsc22d3), MKP1 (also known as dual-specificity phosphatase 1 or *Dusp*), IRAK-M (also know IRAK3- interleukin 1 receptor associated kinase 3), AKT 1 (also know AKT- serine threonine kinase 1) and p65 (also know RELA proto-oncogene, NF-kB subunit). The primer pairs for the gene sequences of *M*. *auratus* GADPH (F: GAC TCA TGA CCA CAG TCC ATG C; R: AGA GGC AGG GAT GAT GTT CTG) and p65 (F: GAA GAA GCG AGA CCT GGA GCA A; R: GTT GAT GGT GCT GAG GGA TGC T) as well as *Rattus norvegicus* IRAK-M (F: GCG TGG AGT GTT GCA AGC T; R: TCC ACA ACC GCG GAA ATT C), GILZ (F: CCG GCA ACC CGA ATC A; R: TGA TAG ACC GCC ACC TCC AT), MKP1 (F: CCT GTA CCT GGG AGT GCT T; R: CCC AAG GCG TCG AGC ATA T), and AKT (F: TCA CCT CTG AGA CCG ACA CC; R: ACT GGC TGA GTA GGA GAA CTG G) were used (Applied Biosystems, USA).

## Results

### Macroscopic and histopathological analysis

**Fig 1:**
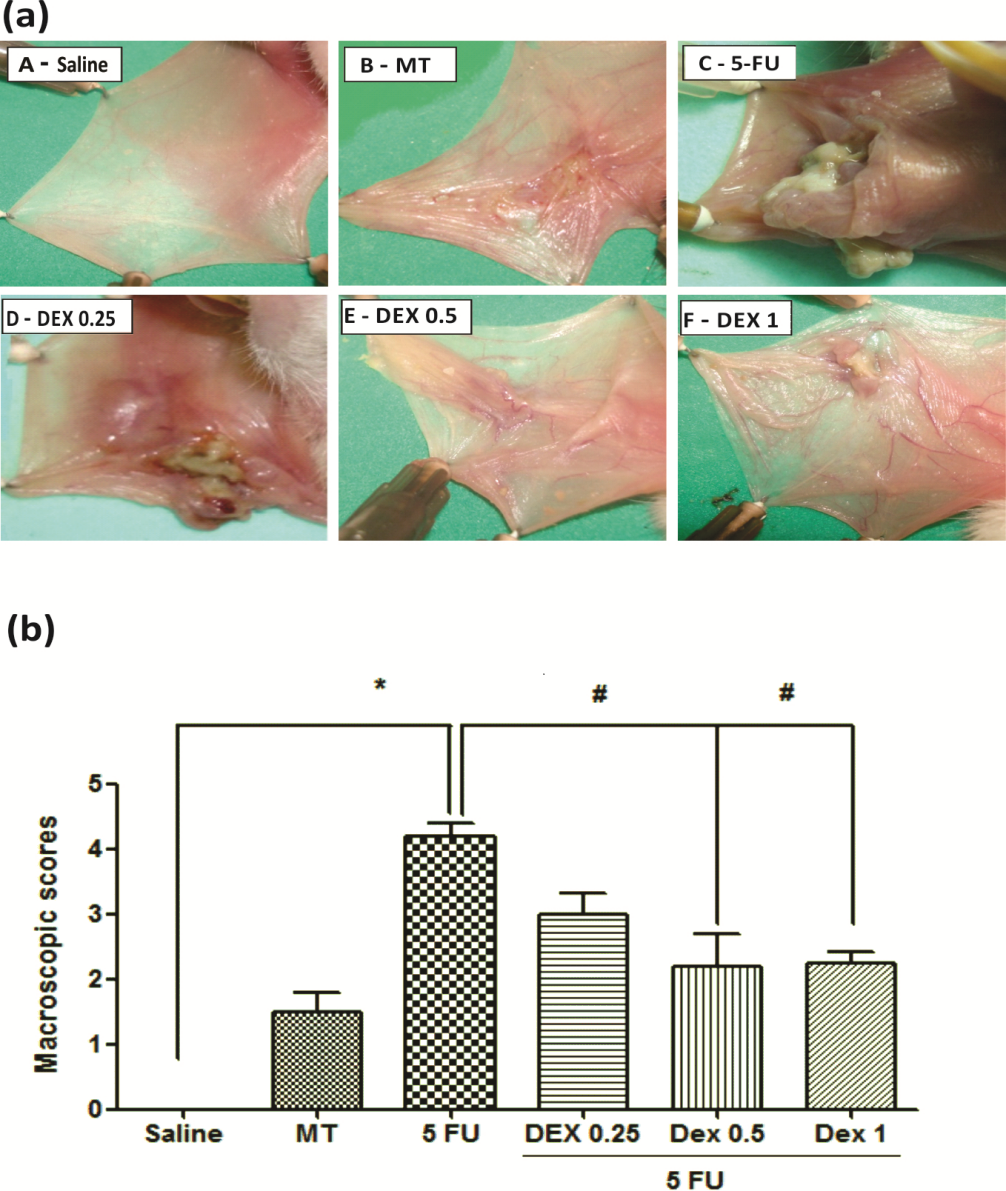
Dexamethasone (DEX) improved the macroscopic analysis (a) and scores (b) of the oral mucosa of hamsters with oral mucositis (OM) induced by 5-fluorouracil (5-FU) and MT. The saline group consisted of normal animals without OM induction (A). The mechanical trauma (MT) group received excoriations in the jugal mucosa, without 5-FU treatment (B). The 5-FU group received 5-FU, was subjected to MT, and was treated with saline i.p. (C). The DEX groups received 5-FU, were subjected to MT, and received DEX i.p. at one of the three different doses 0.25 (D), 0.5 (E), or 1 mg/kg (F). Scores are represented as the median ± standard error of the mean (*n* = 5). *p < 0.05 vs. the saline group, #*p* < 0.05 vs. the 5-FU group (Kruskal–Wallis test and Dunn’s multiple comparison test).

**Fig 2:**
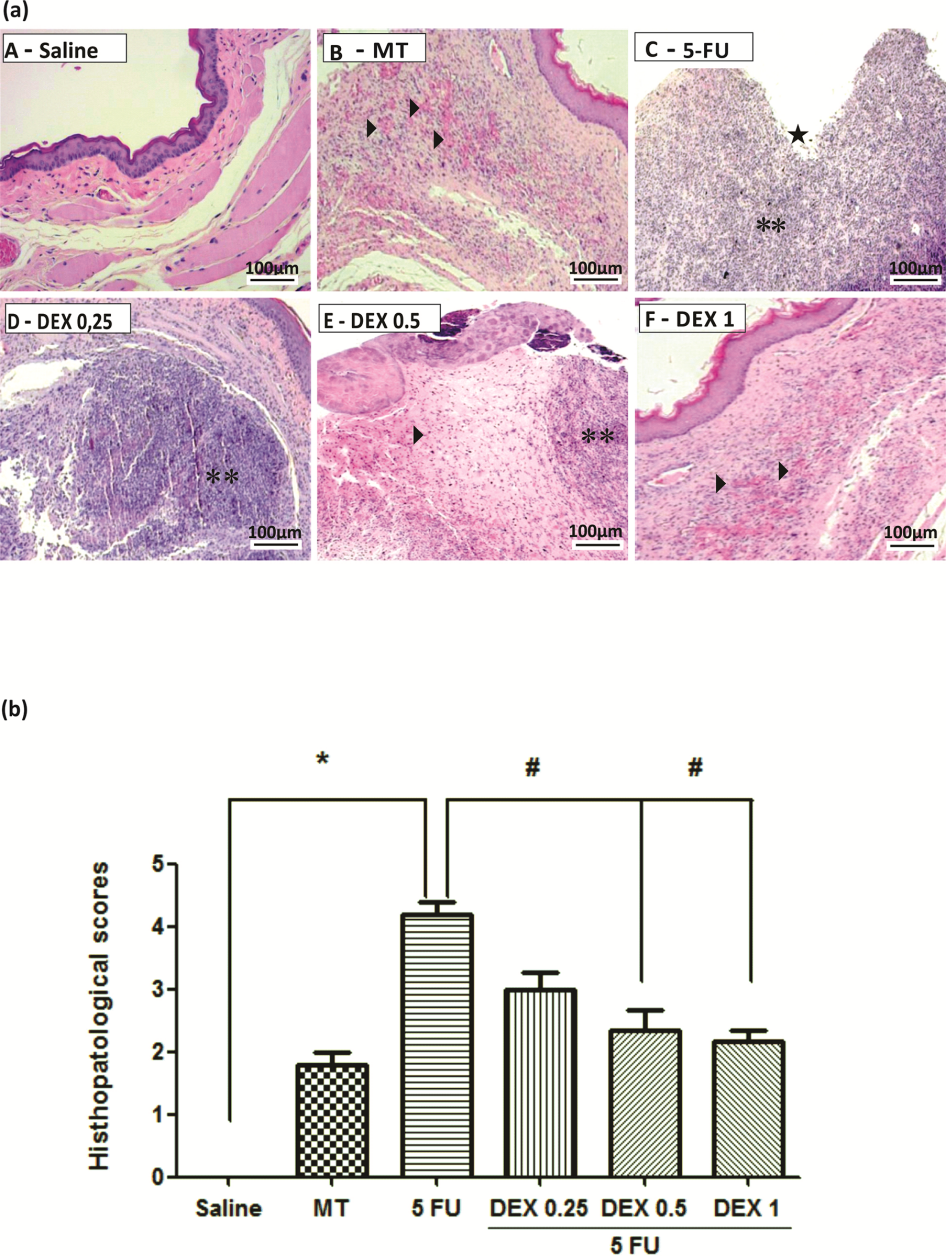
Histopathological analysis (a) and scores (b) of the oral mucosa of hamsters with oral mucositis (OM) induced by 5-fluorouracil (5-FU). The saline group consisted of normal animals without OM and without mucosal alterations (A). The mechanical trauma (MT) group received excoriations in the jugal mucosa, without 5-FU treatment (B). The 5-FU group received 5-FU, was subjected to MT, and was treated with saline i.p.; this group had changes in the jugal mucosa (C), with the presence of ulcers (star) and inflammatory infiltration (two asterisks). The dexamethasone (DEX) groups received 5-FU, were subjected to MT, and received DEX i.p. at one of three different doses: 0.25 mg/kg (D), 0.5 mg/kg (E), or 1 mg/kg (F) with mild hyperemia (an arrow) and absence of ulcers. Scores are represented as the median ± standard error of the mean (*n* = 5). *p < 0.05 vs. the saline group, #*p* < 0.05 vs. the 5-FU group (Kruskal–Wallis test and Dunn’s multiple comparison test).

The jugal mucosa of the saline group was completely healthy (Figs 1A, score of 0 and 2A, score of 1). The MT animals showed the presence of erythema, no evidence of mucosal erosion (Fig 1B, score of 1), discrete vasodilatation, areas of re-epithelialization, discrete cell infiltration, and absence of hemorrhage, edema, ulcers, and abscesses (Fig 2B, score of 2). Untreated animals with OM (the 5-FU group) had ulcers in about 50% of the area of the jugal mucosa (Fig 1C, score of 4, p < 0.05), severe vasodilatation, and inflammatory infiltration with neutrophils, abscesses, and extensive ulcers (Fig 2C, score of 4, p < 0.05), compared to the saline group. The DEX groups had changes in the jugal mucosa induced by 5-FU and MT, and they (0.5 or 1 mg/kg DEX) had erythema, vasodilatation, and superficial erosion (Fig 1E and 1F; score of 2, p < 0.05) as well as discrete vasodilatation, areas of re-epithelialization, discrete cell infiltration, and absence of hemorrhage, edema, ulcers, and abscesses (Fig 2E and 2F; score of 2, p < 0.05), compared to the 5-FU group. DEX 0.25 mg/kg does not have significant alterations, compared to the 5-FU group.

### Cytokine quantification

**Fig 3:**
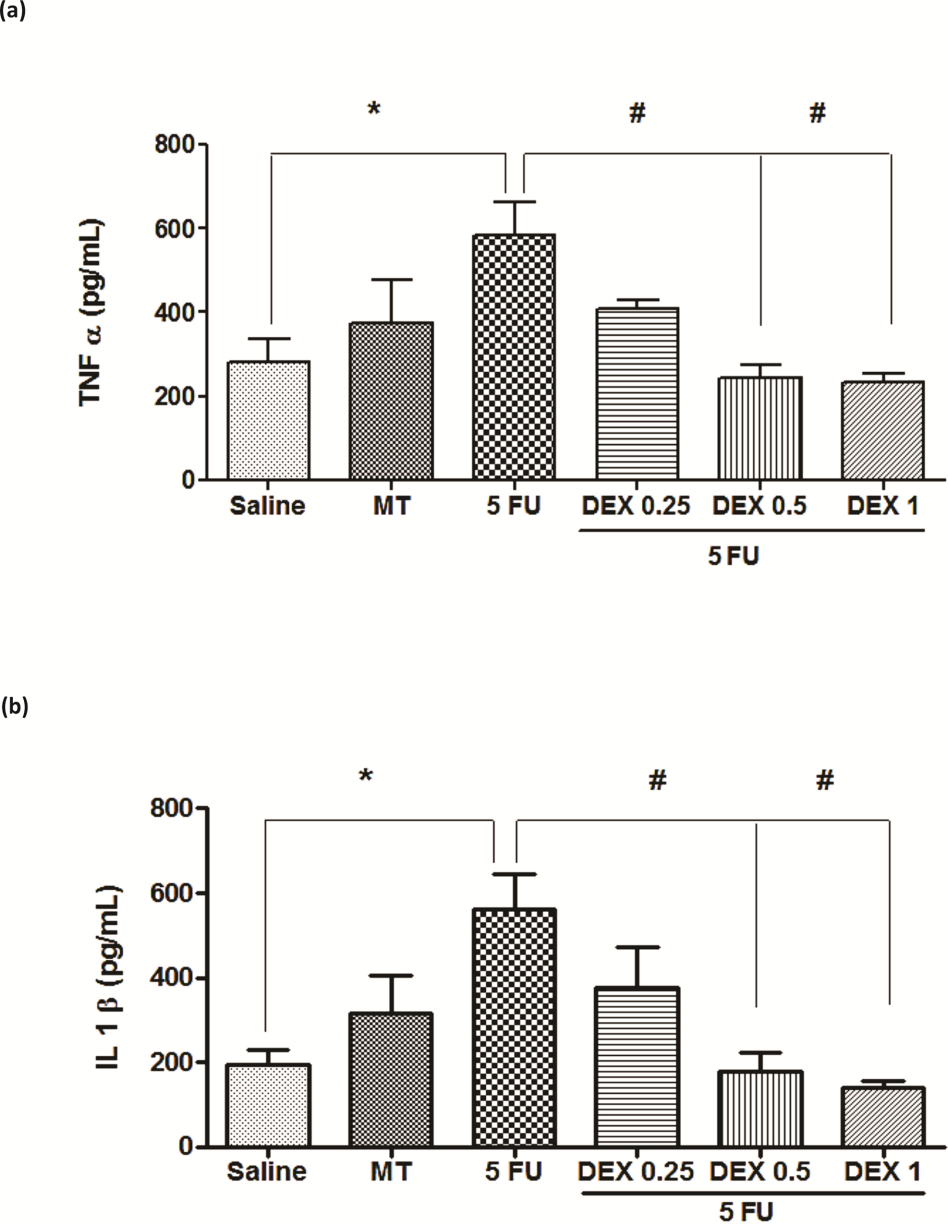
Tumor necrosis factor (TNF)-α (a) and interleukin (IL)-1β (b) cytokines in the oral mucosa of hamsters with oral mucositis (OM). The saline group consisted of normal animals without OM. The mechanical trauma (MT) group consisted of hamsters receiving excoriations in the oral mucosa, without 5-fluorouracil (5-FU) treatment. The 5-FU group received 5-FU, was subjected to MT, and was treated with saline i.p. The dexamethasone (DEX) groups received 5-FU, were subjected to MT, and received DEX i.p. at one of three different doses (0.25, 0.5, or 1 mg/kg). The results are presented as the mean ± standard error of the mean (n = 5). *p < 0.05 vs. the saline group, #*p* < 0.05 vs. the 5-FU group (analysis of variance with Tukey’s post-test).

The 5-FU group had high levels of the cytokines TNF-α (Fig 3A, p < 0.05) and IL-1β (Fig 3B, p < 0.05), compared to the saline group. DEX at a dose of 0.5 or 1 mg/kg reduced the cytokine levels of TNF-α (Fig 3A, p < 0.05) and IL-1β (Fig 3B, p < 0.05), compared to the 5-FU group (hamsters with OM and without treatment). DEX at a dose of 0.25 mg/kg did not prevent the increase in inflammatory cytokine levels induced by OM.

### Immunohistochemistry for MMP-2, COX-2, and TGF-β

**Fig 4:**
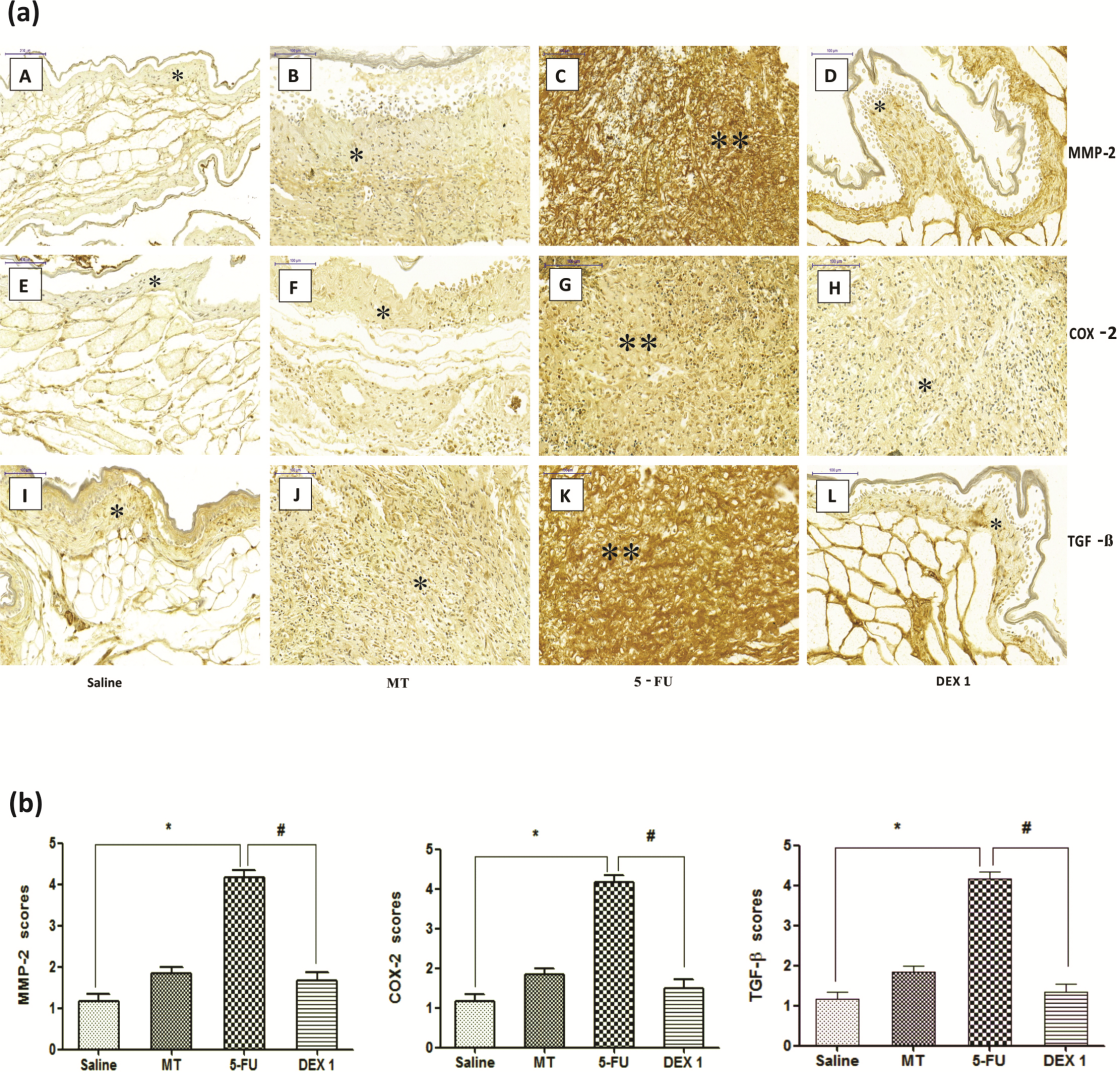
Immunohistochemistry (a) and scores (b) for matrix metalloproteinase-2 (MMP-2), cyclooxygenase-2 (COX-2), and transforming growth factor beta (TGF-β). The saline group (A, E, and I) and the mechanical trauma (MT) group (B, F, and J) had little immunostaining. The 5-fluorouracil (5-FU) group had intense labeling in the jugal mucosa for MMP-2 (C), COX-2 (G), and TGF-β (K), compared to the saline group. Dexamethasone (DEX, 1 mg/kg) reduced the immunostaining for MMP-2 (D), COX-2 (H), and TGF-β (L), compared to the 5-FU group. Poor immunoblotting (an asterisk) and intense immunostaining (two asterisks). Scores are represented as the median ± standard error of the mean (*n* = 5). *p < 0.05 vs. the saline group, #*p* < 0.05 vs. the 5-FU group (Kruskal-Wallis test and Dunn’s multiple comparison test).

The jugal mucosa of the 5-FU group showed intense immunostaining for MMP-2 (Fig 4A–4C and 4B, score of 4, p < 0.05), COX-2 (Fig 4A–4G and 4B, score of 4, p < 0.05), and TGF-β (Fig 4A–4K and 4B, score of 4, p < 0.05), compared with the saline group. On the contrary, the DEX (1 mg/kg) group showed poor immunostaining for MMP-2 (Fig 4A–4D and 4B, score of 2, p < 0.05), COX-2 (Fig 4A–4H and 4B, score of 2, p < 0.05), and TGF-β (Fig 4A–4L and 4B, score of 2, p < 0.05), compared with the 5-FU group.

### Immunofluorescence for MIF, NFκB p65, Smad 2/3 phosphorylated and Smad 2/3

**Fig 5:**
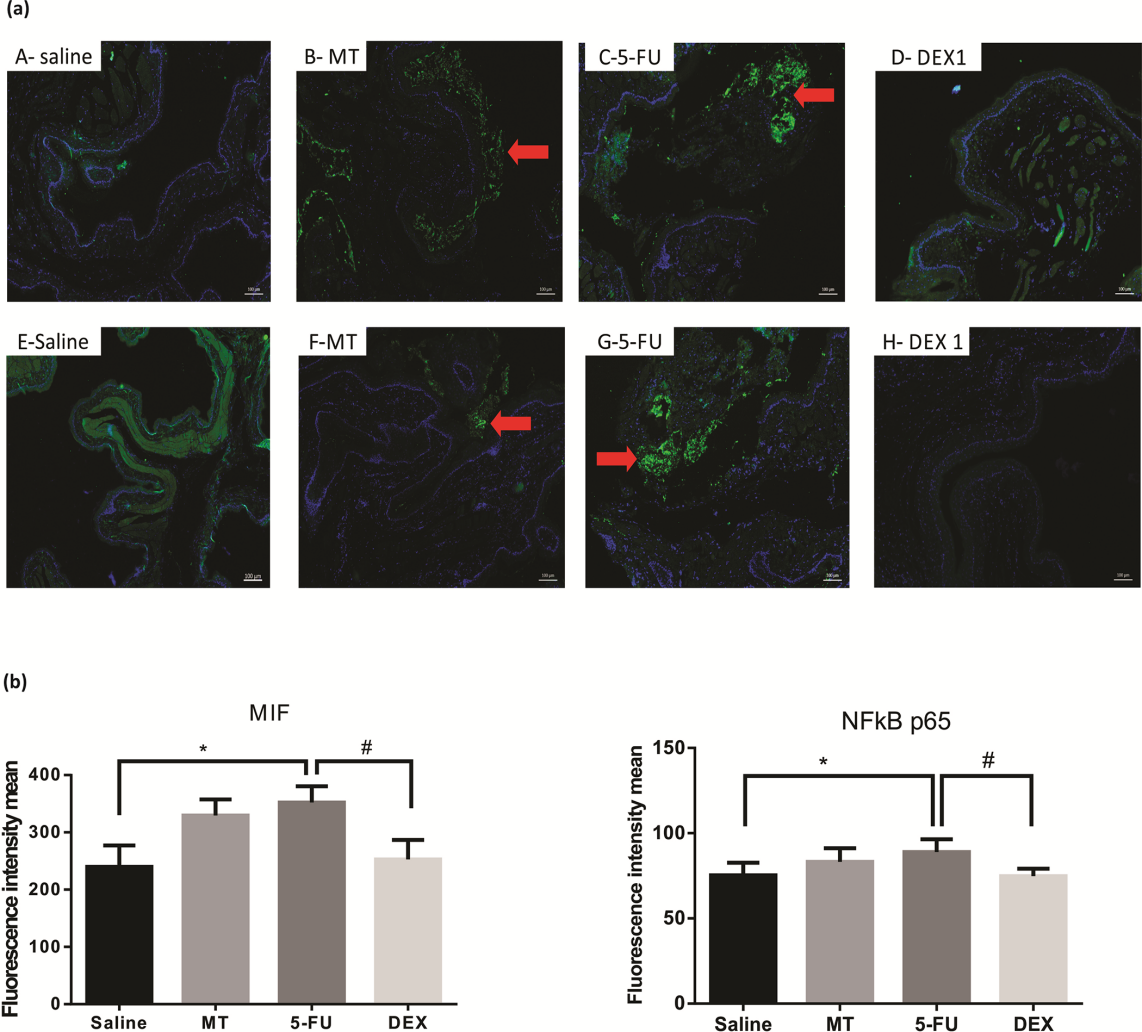
Immunofluorescence for MIF and NFκB p65 (a) and mean of densitometric analysis (b). The 5-fluorouracil (5-FU) group (C and G) had higher green labeling than in the mechanical trauma (MT) group (B and F) or the saline group (A and E) (*p* <0.05; n = 5). DEX 1 mg/kg group (D and H) had a reduced immunoreactivity as compared to the 5-FU group (n = 5; *p < 0.05 vs. the saline group, #*p* < 0.05 vs. the 5-FU group; (analysis of variance with Tukey’s post-test).

**Fig 6:**
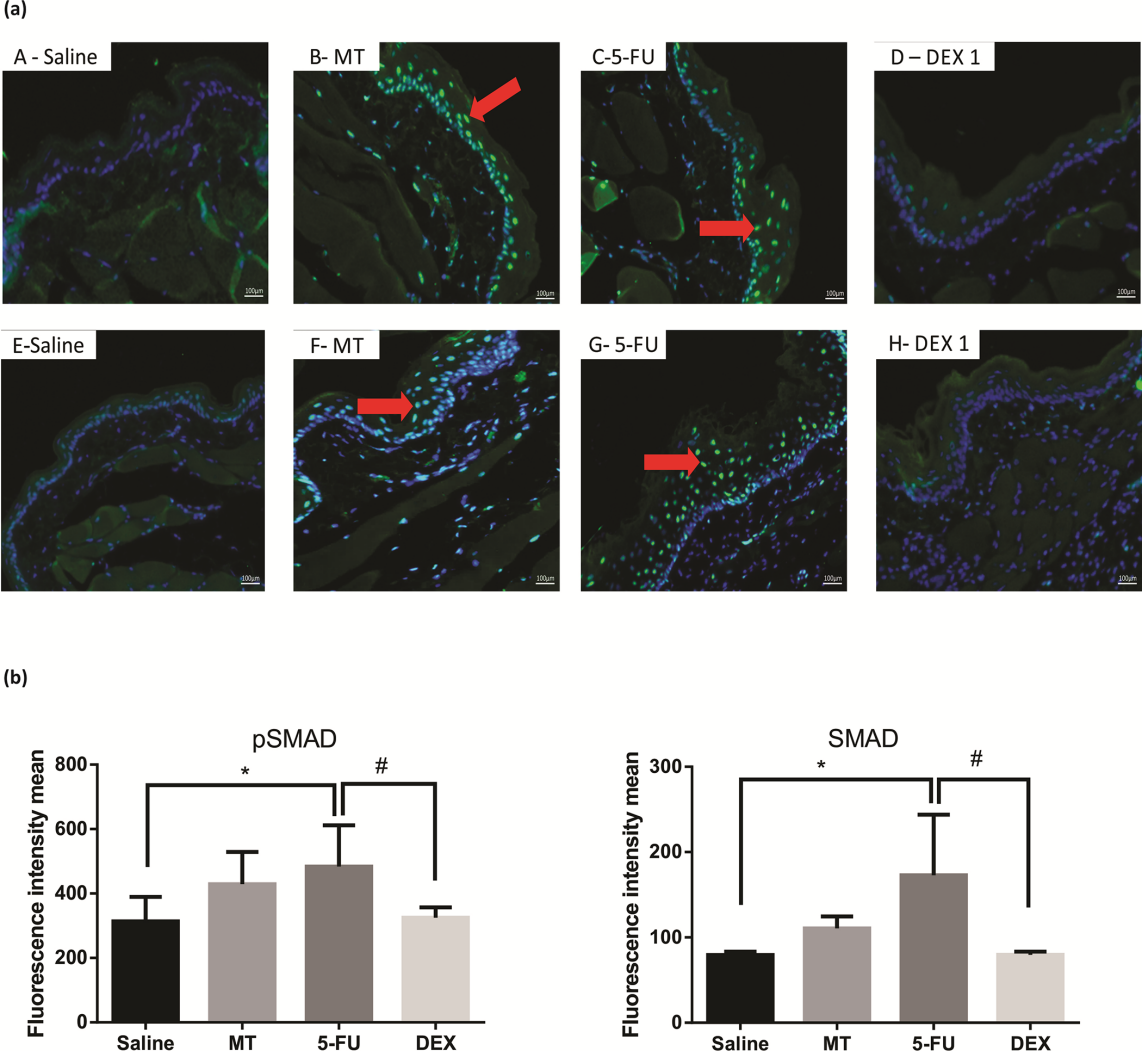
Immunofluorescence for pSmad 2/3 and Smad2/3 (a) and mean of densitometric analysis (b). The 5-fluorouracil (5-FU) group (C and G) had higher green labeling than in the mechanical trauma (MT) group (B and F) or the saline group (A and E) (*p* <0.05; n = 5). DEX 1 mg/kg group (D and H) had a reduced immunoreactivity as compared to the 5-FU group (n = 5; *p < 0.05 vs. the saline group, #*p* < 0.05 vs. the 5-FU group; (analysis of variance with Tukey’s post-test).

Cellular MIF-1, NFκB p65, pSmad 2/3, Smad2/3 labeling (green) was higher and diffuse in the 5-FU group than in the MT and saline groups (Fig 5A and 5B; Fig 6A and 6B respectively; *p* < 0.05). The DEX (1 mg/kg) group had a reduced immunoreactivity for MIF-1, NFκB p65, pSmad 2/3, Smad2/3, compared to the 5-FU group (Fig 5A and 5B; Fig 6A and 6B respectively; *p* < 0.05).

### qPCR for IRAK-M, GILZ, MKP1, AKt, and NFκB p65

**Fig 7:**
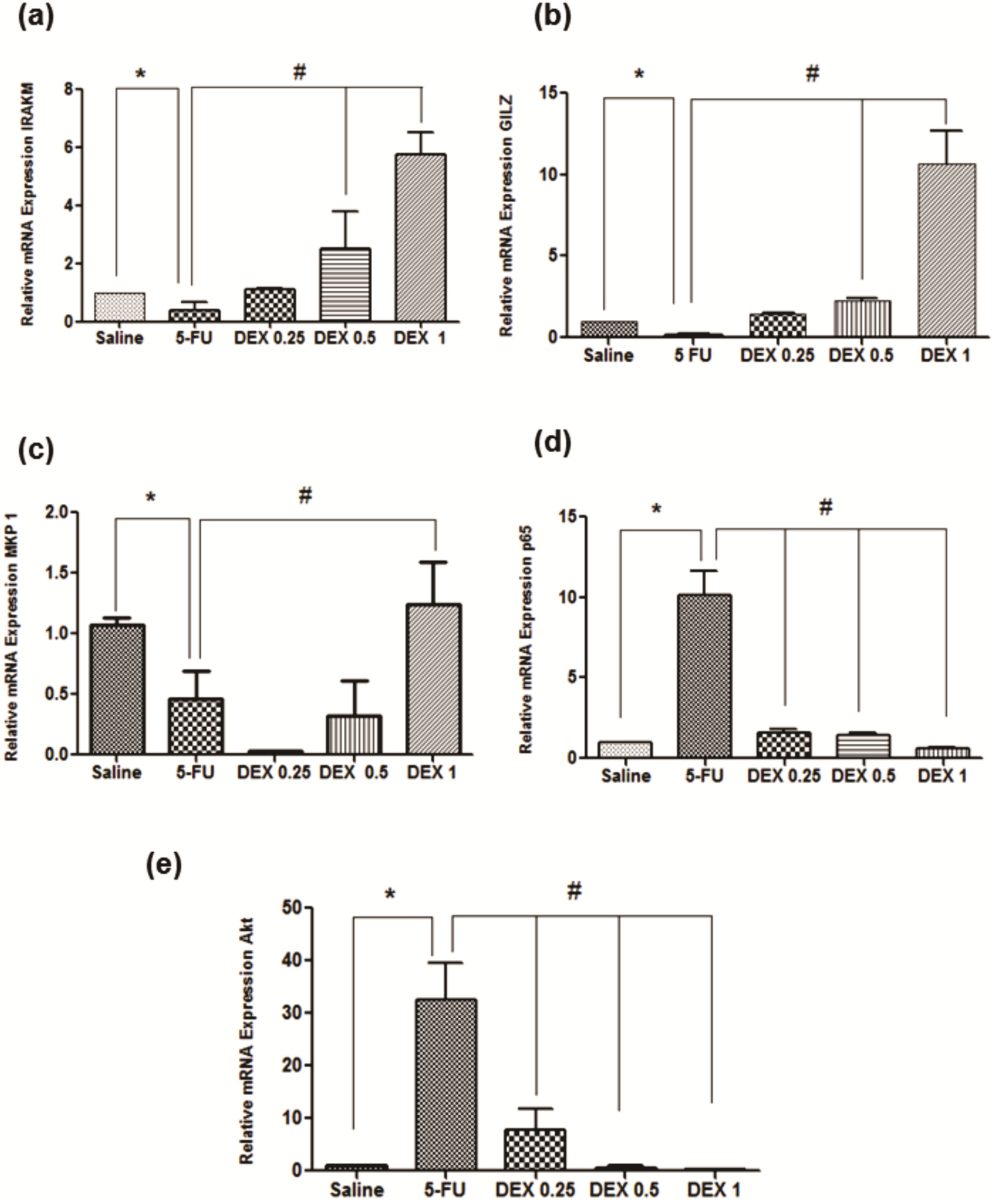
Real-time polymerase chain reaction for interleukin-1 receptor-associated kinase 3 (IRAK-M) (a), glucocorticoid-induced leucine zipper (GILZ) (b), MAPK phosphatase 1 (MKP1) (c), nuclear factor NFκB p65 (d), and serine threonine kinase (AKt) (e). 5-Fluorouracil (5-FU) decreased IRAK-M, GILZ, and MKP1 gene expression as well as increased NFκB p65 and AKt gene expression, compared to the saline group. Dexamethasone (DEX, 1 mg/kg) increased IRAK-M, GILZ, and MKP1 gene expression as well as reduced NFκB p65 and AKt gene expression, compared to the 5-FU group (n = 5; *p < 0.05 vs. the saline group, #*p* < 0.05 vs. the 5-FU group; analysis of variance with Tukey’s post-test).

The 5-FU group had a reduced mRNA expression of IRAK-M, GILZ, and MKP1, compared to the saline group (Fig 7A–7C; p < 0.05), and an increased mRNA expression of NFκB p65 and AKt (Fig 7D–7E; p < 0.05). DEX (1 mg/kg) increased the mRNA expression of IRAK-M, GILZ, and MKP1 and reduced the mRNA expression of NFκB p65 and AKt, in relation to the 5-FU group (Fig 7A–7E, p < 0.05).

## Discussion

In the present study, according to the macroscopic and histopathological analysis, DEX (0.5 or 1 mg/kg) improved lesions of the oral mucosa of hamsters submitted to OM induced by 5-FU. These results are corroborated by a previous study demonstrating that DEX promoted re-epithelialization of the jugal mucosa and reduced inflammatory infiltration on day 10 of the experimental OM model [10].

The pathophysiology of OM is complex, with several signaling pathways participating in the process, such as NFκB signal transduction pathway [4]. The classical pathway of NFκB is formed predominantly by the two dimers p50/p65, which are inactivated in the cytoplasm; when activated, they migrate to the cell nucleus, thus interfering with gene transcription [24]. In OM, NFκB enhances the expression of the proinflammatory cytokines TNF-α and IL-1β as well as induces the expression of COX-2, TGF-β, and MMPs. Proinflammatory cytokines participate in the primary damage to the connective tissue and activate the signaling of the mucosal epithelium, thus amplifying the lesion [3].

In this study, DEX downregulated the relative gene expression and protein expression of NFκB p65 and reduced the levels of proinflammatory cytokines, such as TNF-α and IL-1β, thus contributing to explain the reduction of ulceration and inflammation in the jugal mucosa of animals subjected to 5-FU-induced oral mucositis, as observed by the macroscopic and histopathological analysis. In addition, DEX (1 mg/kg) treatment reduced the oral mucosal expression of COX-2 and MMP-2, a zinc-dependent endopeptidase, which activation contributes to the extracellular matrix degradation [25]. It is well established that NFκB promotes COX-2 expression, which is critical for the production of proinflammatory prostaglandins [5]. It has been suggested that prostaglandins induce MMP expression and activation. A pilot clinical trial showed that specific MMP inhibition seems effective to treat patients under chemotherapy, radiotherapy or both and suffering from mild to severe mucositis [25].

It has been previously reported that the activity of MMPs is regulated by TGF-β. In the current study, DEX (1 mg/kg) treatment reduced the OM-induced activation of TGF-β signaling transduction pathways, observed by the reduction of immunoexpression of TGF-β, Smad 2/3 and phosphorylated Smad 2/3 (pSmad 2/3) in the jugal tissues of animals submitted to oral mucositis. TGF-β, produced by keratinocytes and macrophages, acts through type I or II receptors via Smad 2/3 protein signaling [26]. In the oral mucosa, TGF-β promotes cell apoptosis and activates NFκB; while in keratinocytes, at high concentrations, it interferes with mucosal healing [27]. Substances that negatively interfere with the TGF-β signaling pathway have a protective effect on the oral mucosa, such as the nuclear protein Smad 7 [28]. Similar to the findings of this study, others have demonstrated that DEX inhibits TGF-β signaling [29], most probably by preventing the NFκB activation. In fact, a key mechanism in the antiinflammatory action of glucocorticoids is repression of NFκB signaling pathway, thus interfering with the expression of NFκB-regulated proiinflammatory genes, including TGF-β and inflammatory cytokines, such as TNF-α [30]. In accordance, in the present study, the anti-inflammatory effects of DEX are associated with reduced mRNA and protein expression of NFκB subunit p65 and decreased levels of TNF in the oral mucosa of animals submitted to 5-FU-induced OM. On the other hand, TNF-α is one of the most potent physiological inducers of NFκB [31], contributing to amplify the inflammation and tissue damage. In view of this, we speculate that dexamethasone suppressed the transcription and the production, by epithelial and connective tissue cells, of inflammatory cytokines, mainly IL-1β and TNF-α, preventing the NFκB-transactivation.

In addition, DEX regulated other signaling pathways that might contribute for its protective effects on 5-FU-induced OM. DEX (1 mg/kg) increased the gene expression of IRAK-M and GILZ. IRAK-M (IRAK3) is a member of the IRAK family, which regulates toll-like/IL-1 receptors. IRAK-M inhibits IRAK1, which is responsible for activating the NFκB pathway [32]. Thus, DEX causes an increase of IRAK-M expression, which suppresses NFκB-mediated inflammation. Similarly, others have demonstrated that DEX increases IRAK-M signaling in inflammation models [13].

Glucocorticoids induce the expression of GILZ, a glucocorticoid-induced leucine zipper, which is a member of the TSC-22 protein family that is important for mediating anti-inflammatory activity. GILZ inhibits the NFκB and AKt pathways [12]. AKt is a serine threonine kinase that activates NFκB and stimulates the production of proinflammatory cytokines. In this work, DEX (1 mg/kg) increased the gene expression of GILZ and reduced the gene expression of AKt [33]. It has been suggested that GILZ inhibits Akt by binding to activated Ras [34]. GILZ also interacts with mammalian rapamycin target pathway 2 complex (mTORC2), inhibiting Akt phosphorylation [35]. Thus, GILZ regulates important inflammatory pathways, which might contribute to the protective effect of DEX observed in this current work. Accordingly, 5-FU downregulated IRAK-M and GILZ gene expression in the present study. Protein-expression studies, however, are necessary to confirm the participation of IRAK-M, GILZ and Akt in the mechanisms associated with the protective effects of DEX against 5-FU-induced OM.

In the present study, DEX (1 mg/kg) reduced immunoreactivity for MIF. MIF is a cytokine that is produced by the anterior pituitary gland and acts on the CD74-CD44 receptor, activating pathways such as mitogen-activated protein (MAP) kinase and phosphoinositide-3-kinase and enhancing the expression of proinflammatory genes [36]. MIF regulates the anti-inflammatory and immunosuppressive activity of glucocorticoids, notably by reducing the expression of MKP1 [15]. MKP1 is a phosphatase that inhibits TNF-α-induced inflammation and dephosphoryl kinases critical for the inflammatory process, such as p38 MAP kinase and c-Jun N-terminal kinase [14]. In this study, the 5-FU group had increased MIF expression and reduced MKP1 expression. DEX reduced MIF expression and increased MKP1 expression, proposing that MIF and MKP1 participate in the protective effect of DEX on OM lesions.

**Fig 8:**
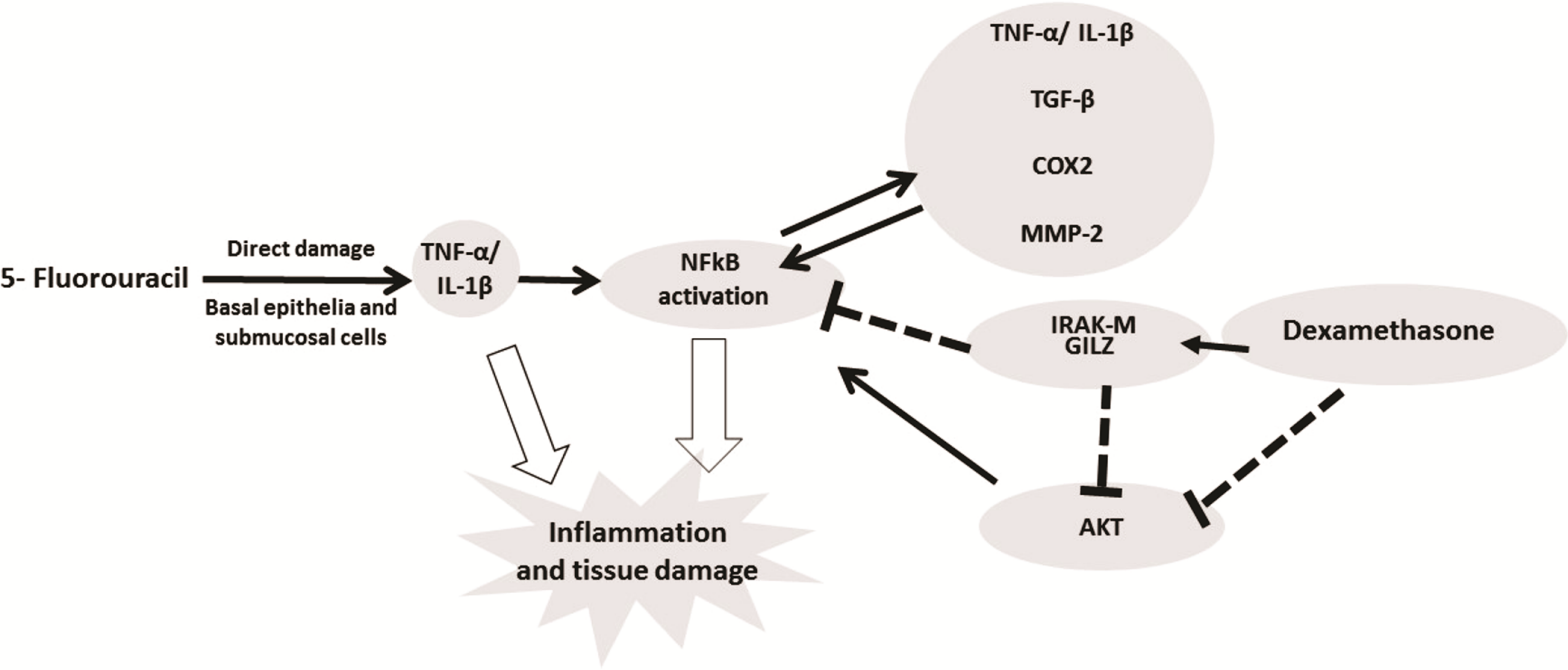
Signaling pathways of dexamethasone modulation in oral mucositis induced by 5-fluorouracil (5-FU). 5-FU activates NFkB which induces expression of pro-inflammatory cytokines (IL-1 β, TNF-α) that promote tissue damage and inflammation in the oral mucosa. Dexamethasone downregulated the NFkB by increasing the expression of IRAKM and GILZ and decreased gene expression of AKt. Dexamethasone interfering with NFkB decreases IL-1 β, TNF-α, TGF-β, COX2 and MMP2 by improving oral mucositis. (NFκB—the nuclear transcription factor kappa B; GILZ- glucocorticoid-induced leucine zipper; IRAK-M—interleukin-1 receptor-associated kinase; AKt- serine threonine kinase).

In conclusion, DEX improved the 5-FU-induced OM lesions by reducing the TNF-α, IL-1β, TGF-β, and MIF cytokine levels; increasing IRAK-M, GILZ, and MKP1 gene expression; and reducing NFκB p65 and AKt gene expression, suggesting that these pathways participate in the protective effect of DEX in OM (Fig 8). However, more studies are needed.
